# Ponatinib-Induced Cerebrovascular Accident (CVA)

**DOI:** 10.7759/cureus.32383

**Published:** 2022-12-10

**Authors:** Farhan Azad, Jiahua Zhang, Clive J Miranda, Matthew Gravina

**Affiliations:** 1 Internal Medicine, University at Buffalo, Buffalo, USA; 2 Internal Medicine, Mercy Hospital St. Louis, St. Louis, USA

**Keywords:** tyrosine kinase inhibitors, ponatinib ph+ acute lymphoblastic leukemia and chronic myelogenous leukemia evaluation, optimizing ponatinib treatment in chronic phase-chronic myelogenous leukemia, bcr-abl, ponatinib

## Abstract

Ponatinib is a highly potent tyrosine kinase inhibitor shown to have excellent outcomes in the treatment of acute and chronic leukemias. Despite its high efficacy, ponatinib has been shown to carry an increased risk for cardiovascular adverse events, not attributable to a known mechanism. We present a case of a patient with Philadelphia chromosome-positive acute lymphoblastic leukemia (ALL) who developed a cerebrovascular condition while receiving maintenance therapy with the lowest treatment dose of ponatinib for a prolonged duration.

## Introduction

Acute lymphoblastic leukemia (ALL) is a proliferation and transformation of lymphoid progenitors in bone marrow, blood, and extramedullary sites. It represents a severe disease in adults, with only 30-40% of patients achieving long-term remission despite chemotherapy [1]. The most common genetic abnormality associated is Philadelphia chromosome (Ph) or BCR-ABL positive, which has been shown to have the worst prognosis [2]. The survival rates improved drastically with the introduction of BCR-ABL tyrosine kinase inhibitors (TKI). Imatinib-based regimens followed by stem cell transplantation (SCT) appeared highly effective, achieving complete response (CR) with decreased recurrence rates [3]. Follow-up studies, however, revealed that nearly 20% of patients on imatinib do not exhibit a complete cytogenic response. Second-generation TKI including nilotinib and dasatinib were designed to be more selective but continued to show resistance to some BCR-ABL mutations. Ponatinib, a potent third-generation TKI, was then developed, and showed activity against both native and most clinically relevant BCR-ABL mutations. It also showed inhibition of T315I, a BCR-ABL mutation often resulting in resistance to second-generation TKI [4].

Further studies show ponatinib and its multi-targeted characteristics give it the ability to inhibit other tyrosine kinases including fibroblast growth factor receptor (FGFR), platelet-derived growth factor receptor (PDGFR), SRC, rearrangement during transfection (RET), KIT, and FLT1 in the treatment of malignancies [5]. In BCR-ABL positive ALL patients, the efficacy data for ponatinib from the Ponatinib Ph+ Acute Lymphoblastic Leukemia and Chronic Myelogenous Leukemia Evaluation (PACE) trial demonstrated promising results, with 41% of patients having a major hematologic response, 47% major cytologic response (MCyR), and 38% complete cytologic response (CCyR) [6]. Despite showing promise, prolonged TKI use has been associated with cerebrovascular accidents (CVAs), myocardial infarction, and peripheral vascular disease [7]. In particular, ponatinib-related vascular adverse events have been reported in 20-40% of patients receiving the standard therapy. Ponatinib has been associated with cardiovascular events and hypertension, although the causes of these events are unclear [8]. It is suspected that ponatinib caused the acute cerebral event in our patient. 

## Case presentation

A 63-year-old African American female with a history of asthma, seasonal allergies, and B-cell ALL diagnosed in 2019 in remission with ponatinib presented with multiple falls at home over a week. Falls were progressively worsening and increasing in frequency. She denied any light-headedness, dizziness, chest pain, or palpitations before her falls. She also denied any head trauma or loss of consciousness. She noted intermittent weakness and heaviness of her right arm and right leg, right facial droop, and slurred speech. Social history was negative for smoking. Vitals revealed a heart rate of 90 beats per minute, blood pressure of 138/80 millimeters of mercury, and temperature of 37.6°C, all of which remained stable throughout. The exam was unrevealing of aphasia, unequal motor strength, or weakness. The calculated National Institutes of Health Stroke Scale (NIHSS) score was zero. No tissue plasminogen activator (tPA) was administered as she presented outside the window for therapy. Her laboratory investigations on admission are shown in Table 1.

**Table 1:** Initial laboratory investigations MCV: Mean corpuscular volume; PT: Prothrombin time; INR: International normalized ratio; PTT: Partial thromboplastin time; GFR: Glomerular filtration rate; BUN: Blood urea nitrogen

Laboratory investigations	Results	Reference Range
Complete blood count		
White blood cells	10.1x10^9^/L	4.0-10.5x10^9^/L
Hemoglobin	14.9 g/dL	12.0-16.0 g/dL
Hematocrit	46.7%	37.0-47.0%
MCV	84.1 fL	80.0-100.0 fL
Platelet count	355x10^9^/L	140x10^9^-400x10^9^/L
Coagulation tests		
PT	10.3 seconds	9.4-12.5 seconds
INR	1.22	0.00-3.50
PTT	32.1 seconds	25.0-35.0 seconds
Basic metabolic panel		
Sodium level	138 mmol/L	133-147 mmol/L
Potassium level	3.9 mmol/L	3.5-5.6 mmol/L
Chloride	106 mmol/L	96-110 mmol/L
Carbon dioxide	25 mmol/L	20-32 mmol/L
Calcium level	8.9 mg/dL	6.3-11.9 mg/dL
GFR	> 60 mL/min/1.73 m2	≥ 60 mL/min/1.73 m2
BUN	9 mg/dL	5-27 mg/dL
Creatinine	0.80 mg/dL	0.40-1.40 mg/dL
Glucose level	128 mg/dL	60-100 mg/dL

Subsequent laboratory investigations are shown in Table 2.

**Table 2:** Subsequent laboratory investigations HDL: High-density lipoprotein; LDL: Low-density lipoprotein; VLDL: Very low-density lipoprotein

Laboratory investigations	Results	Reference Range
Lipid Panel		
Triglycerides	179 mg/dL	30-150 mg/dL
Cholesterol HDL	34 mg/dL	40-60 mg/dL
VLDL Cholesterol (Calculated)	42.0 mg/dL	0.0-30.0 g/dL
Cholesterol LDL (Calculated)	149 mg/dL	0-100 mg/dL
Non-HDL Cholesterol (Calculated)	221 mg/dL	≤ 129 mg/dL
Other tests		
A1c	6.3	4.0-6.0
Troponin	< 0.01 ng/mL	0.00-0.06 ng/mL

**Figure 1:**
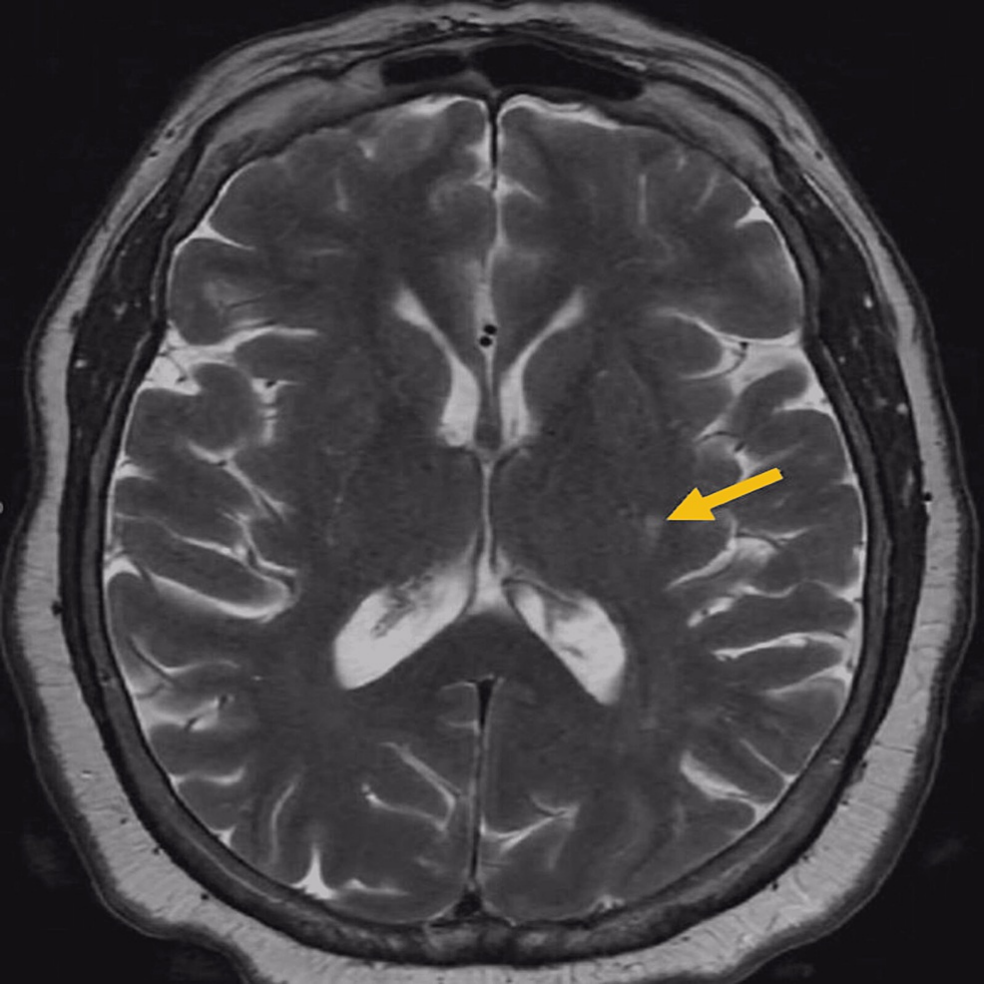
MRI of the brain showing scattered T2 hyperintensities in the subcortical white matter including the left basal ganglia, periatrial region, and insula, and restricted diffusion within the left posterior basal ganglia (arrow)

CT stroke study revealed left M1 occlusion with distal reconstitution, and perfusion deficit in the left middle cerebral artery (MCA) territory. MRI of the brain showed scattered T2 hyperintensities in the subcortical white matter including the left basal ganglia, periatrial region, and insula, and restricted diffusion within the left posterior basal ganglia (Figure 1).

An electrocardiogram revealed that sinus rhythm and troponin levels were normal. Echocardiography showed preserved ejection fraction with no thrombus, vegetation, or tumors. Carotid dopplers revealed less than 50% stenosis bilaterally with patent subclavian and vertebral arteries. The patient was started on aspirin, clopidogrel, and atorvastatin. Oncological history included a diagnosis of BCR-ABL B-cell ALL via bone marrow biopsy showing t(9;22)(q34.1;q11.2), now in remission on ponatinib maintenance therapy. She received ponatinib 30 mg daily for two months, followed by 15 mg daily for 23 months before this acute event. The last dose of ponatinib was taken on the day when symptoms first appeared. Additional prior chemotherapy included three cycles of induction and six cycles of consolidation with high-dose cytarabine (HiDAC), followed by 11 cycles of maintenance therapy with vincristine and prednisone pulses. Ponatinib was held due to a possible contributing factor to the CVA. On day five of hospitalization, the patient was noted to have symptoms of aphasia and right-sided weakness, similar to her presentation at home. Due to the apparent failure of medical treatment, she underwent a superficial temporal artery (STA) to MCA bypass with no complications. A multidisciplinary rehabilitation program was initiated, and the patient was discharged in two weeks in stable condition.

## Discussion

The underlying mechanism of ponatinib-induced increased CVA risk is unclear. While arterial thrombotic events have been noted, platelet involvement is unlikely as ponatinib inhibits platelet activation and aggregation. Comparison of ponatinib with other TKIs show more potent vascular endothelial growth factor receptor (VEGFR) inhibition, reducing the function and viability of endothelial cells (EC) causing vascular toxicity. Given that our patient did not have any prior cardiovascular risk factors, the acute vascular event was likely related to EC inflammation, apoptosis, and dysfunction induced by ponatinib [9].

Multiple trials have explored the viability of using ponatinib in hematological malignancies. PACE was a phase II trial showing the efficacy and safety of ponatinib in patients with pretreated chronic myelogenous leukemia (CML) or Ph-positive ALL resistant to dasatinib or nilotinib. Favorable long-term outcomes were seen, with five-year progression-free survival (PFS) and overall survival (OS) rates at 53% and 73%, respectively. However, the initial report showed cumulative incidence (CI) of arterial occlusive events (AOEs) to be 17.1% with a two-year follow-up. In the five-year follow-up, CI was 25% in the overall population and 31% in chronic phase-CML (CP-CML), contributing to a longer duration of treatment. Of 10 patients who had a first AOE following dose reduction to 15 mg, four had the event within six months compared to 23 months in our patient. Thus, the timing can be variable, and patients should be monitored regularly for hypertension, AOE, and cardiac function to reduce or even discontinue ponatinib as needed [10]. In our case, ponatinib was held during the hospitalization and discontinued at discharge. STA-MCA bypass is a salvage revascularization technique used in treatment-refractory MCA strokes. It involves branches of STA anastomosing to M3/M4 or cortical branches of MCA, bypassing the occluded branches, and improving perfusion in the ischemic area. While not routine, early STA-MCA bypass has been shown to improve neurological and functional outcomes [11]. Our patient showed progressive improvement and was stable for discharge two weeks after the procedure.

The phase III Evaluation of Ponatinib versus Imatinib in Chronic Myeloid Leukemia (EPIC) trial intended to compare the efficacy and safety between ponatinib and imatinib in newly diagnosed CP-CML. It was terminated early due to AOEs with ponatinib therapy. Ponatinib treatment showed 7% AOE compared to 2% in the imatinib-treated group. The proportion of patients with one or more cardiovascular risk factors was higher in the ponatinib arm, although neither PACE nor EPIC trial could propose a mechanism for ponatinib-induced cardiovascular disease formation [12]. It is unclear if our patient developed cardiovascular disease from prolonged ponatinib therapy. While the lipid panel showed mildly elevated LDL-cholesterol, ponatinib therapy does not appear to modify the plasma lipid profile [13].

Dose reduction has been proposed to decrease the AOE risk of ponatinib. The Optimizing Ponatinib Treatment in Chronic Phase-Chronic Myelogenous Leukemia (OPTIC) study showed benefit at all three ponatinib doses (45 mg, 30 mg, 15 mg) and robust long-term survival in all three arms, consistent with the PACE trial. This suggests that the dose reduction strategy did not affect survival. However, the trial revealed that a 45 mg starting dose reduced to 15 mg upon reaching ≤1% BCR-ABL gives the best response while reducing toxicity [14]. Despite the reduced dose of ponatinib, our patient developed an acute vascular event. In absence of other major cardiovascular risk factors, prolonged ponatinib therapy may have predisposed our patient. Further studies are needed to investigate the safe dose and duration of ponatinib treatment while minimizing the risk of AOE.

## Conclusions

Our patient developed a MCA infarct after prolonged ponatinib therapy for more than 20 months. She was on maintenance therapy with the lowest dose of ponatinib. The patient had no cardiovascular comorbidities or prior history of ischemic events predisposing her to this acute event. Other TKI have been associated with arterio-occlusive events, although ponatinib is the most well-known as evidenced by the literature. These patients need regular follow-up and management of existing cardiovascular conditions. Patients on ponatinib therapy should also be made aware of the potential benefits and side effects of the medication.
